# Current News and Opinion

**Published:** 1883-09

**Authors:** 


### Current News and Opinion.

### AMERICAN ASSOCIATION FOR THE ADVANCEMENT OF SCIENCE.

The meeting of this Society for the year 1883, under the Presidency of Prof. C. A. Young, of Princeton, was held at Minneapolis, Minn., from August 15 to August 22. The Association is divided into nine sections: A, Mathematics and Astronomy; B, Physics; C, Chemistry; D, Mechanical Science; E, Geology and Geography; F, Biology; G, Histology and Microscopy; H, Anthropology; I, Economic Science and Statistics; and thus it insures to its members all possible opportunities to meet those who are following the same lines of research. The majority of the sections held active and interesting sessions, particularly those of Geology, Biology, Anthropology, and Physics, while Mechanical Science and Economic Science consumed the largest amount of time in organizing and preparing for work, of which there was little done that was worth mentioning. The poorest section of all is that of Microscopy and Histology, and for good reasons. Unless the term

Microscopist includes all those mere manipulators of the instrument who make a toy of it, and exhibit indifferent objects for the inspection of the curious, there would seem to be no necessity for the existence of such a section. If we investigate with the microscope animal or vegetable parasites, high or low forms of life, the work properly belongs in Biology. If sections of stone or fossil be studied, we are working in Geology. There is no place for microscopy, unless it be in the study of the instrument itself, for, properly used, it is but a tool with which to investigate certain fields of science. It is little wonder, then, that scientists who are pursuing their work through the microscope should prefer membership in the American Society of Microscopists.

The citizens of Minneapolis extended unusual hospitalities to the members of the Society, and many of the latter went to their eastern homes with ideas of western life somewhat modified.

The next annual meeting will be held in Philadelphia, with Prof. J. P. Leslie, the State Geologist of Pennsylvania, as President. This meeting promises to be of special interest, as the British Association is expected to meet in Montreal in 1884, and the American scientists will extend a cordial invitation to their British brethren to come from there to Philadelphia.

---

### AMERICAN SOCIETY OF MICROSCOPISTS.

The sixth annual session of this Association was held in the city of Chicago, commencing Tuesday, Aug. 7th, and continuing four days. It was unfortunate that it was called for the same date as that of the meeting of the American Dental Association, as a number of gentlemen hold membership in both. The Society is young, but it has made very rapid progress in the scientific world. The late meeting was a very successful one, whether we consider the number of members in attendance, the zeal and enthusiasm manifested, the social enjoyment and fraternal feeling which marked the occasion, or the amount of real commendable work done.

Prof. Albert McCalla, of Fairfield, Iowa, was the President, and Prof D. S. Kellicott, of Buffalo, N. Y., was Secretary.

There were more papers presented than there was time to properly consider, and several of them were merely read by title and referred. Some of the essays were of unusual merit, particularly those of Prof. Rogers, of Cambridge.

One good thing done was the adoption of a standard micrometer, which will, it is hoped, reduce the confusion that has heretofore reigned supreme in microscopical measurements.

The Royal Microscopical Society of England, having extended recognition by the adoption of a resolution making the President of the American Society an ex-officio fellow, the Chicago meeting returned the compliment by electing the President of that Society to honorary membership.

Hon. J. D. Cox, of Ohio, a well-known and enthusiastic microscopist, was elected President for the ensuing year, and Prof. Kellicott remains the very efficient Secretary. The next meeting will be held in Rochester, N. Y.

---

### NOVEL LARYNGOSCOPE.

Dr. Thomas Dimock, in the *Therapeutic Gazette*, details a new method of examining the throat. Place the patient before a good light, depress the tongue and request him to yawn. The larynx immediately rises up, the velum palati is lifted and the anterior and posterior pillars widened, exposing the back of the tonsils and the pharynx. The nose should be held to compel breathing through the mouth.

---

### NEW COLLEGE.

A new medical college has been organized in Buffalo. We ought to be thoroughly taught, medically, for there are now three colleges in the city. The faculty of the new institution comprises some excellent names, while there are other chairs that will be occupied—not filled. Upon the whole it is a better faculty than one would have thought it possible to organize in Buffalo, outside of those engaged in other schools.

### TRANSACTIONS OF THE DENTAL SOCIETY OF THE STATE OF NEW YORK.

The undersigned would like a dozen or more copies of the transactions of 1869–70; also a few of 1871–2, and 1873–4. If desired, will give in exchange copies of 1875–6, or 1877–8, or 1882–3, which last will be issued on or about October 1st of the present year. Send transactions to, or address,

J. EDWARD LINE, Secretary,
Rochester, N. Y.

---

### PAINTING TEETH.

Sometimes it is difficult to obtain artificial teeth having just the shade desired. Prof. T. L. Buckingham recommends that in such cases a tooth be painted the desired color with common oil paints, and that then it be heated in a common crucible and the tints thus burned in. A little practice will, he says, enable one to obtain any unusual color, and thus correctly to match any of the natural teeth.

---

### DENTAL LEGISLATION.

Michigan now has an excellent dental law, and that State henceforth ceases to be a harbor of refuge for the expelled quacks of other States. Illinois, too, has been added to the number, and so the work goes on. We are not of those who believe that dentists can be made by legal enactment, but a certain class of so-called dentists can and should be un-made by it.

---

### RESIGNED.

Prof. John C. Dalton, so long and favorably known as a teacher, has resigned his professorship in the College of Physicians and Surgeons of New York, on account of ill health. The thousands of admirers of Dr. Dalton will sincerely hope that this does not mean a relinquishment of the active part in professional matters which he has always taken.

### NEW BOOK.

Surgeon-General Wales is preparing an original work upon Diseases of the Rectum, etc. The readers of the INDEPENDENT PRACTITIONER know how thorough the work will be, from a perusal of his article on "Abscesses of the Ano-Perineal Region," contributed to and printed in the May number.

---

### THE OHIO JOURNAL.

Dr. JUNIUS E. CRAVENS has joined the editorial staff of the *Ohio State Journal of Dental Science*, and has opened a new department, "Varieties," in which will be found his contributions. His initial, in the August number, is both spicy and instructive.

---

### EDITORIAL CHANGE.

Dr. L. S. McMurtry has retired from the Associate Editorship of that excellent weekly, *The Louisville Medical News*, and Dr. H. A. Cottrell, formerly associated with the *Journal*, succeeds him.

---

### CALLED.

Dr. A. J. C. Skeene has been added to the faculty of the New York Post Graduate course. His department will be Gynæcology.

