# Erroneous

**Published:** 1883-10

**Authors:** 


### ERRONEOUS.

In the paragraph entitled "Painting Teeth," in the Sept. No. of this journal, we stated that Dr. Buckingham recommended common oil paints for burning in, to impart any special tints to artificial teeth. We should have said—vitrifiable paint, like that used for painting on china, tiles, etc. It may be obtained at the art furnishing stores. We are obliged to Dr. W. H. Trueman for the correction.

